# A community jury study exploring the public acceptability of using risk stratification to determine eligibility for cancer screening

**DOI:** 10.1111/hex.13522

**Published:** 2022-05-08

**Authors:** Rebecca A. Dennison, Rachel A. Boscott, Rae Thomas, Simon J. Griffin, Hannah Harrison, Stephen D. John, Sowmiya A. Moorthie, Stephen Morris, Sabrina H. Rossi, Grant D. Stewart, Chloe V. Thomas, Juliet A. Usher‐Smith

**Affiliations:** ^1^ Primary Care Unit, Department of Public Health and Primary Care University of Cambridge Cambridge UK; ^2^ School of Clinical Medicine University of Cambridge Cambridge UK; ^3^ Institute for Evidence‐Based Healthcare Bond University Gold Coast Queensland Australia; ^4^ Department of History and Philosophy of Science University of Cambridge Cambridge UK; ^5^ PHG Foundation University of Cambridge Cambridge UK; ^6^ Department of Oncology University of Cambridge Cambridge UK; ^7^ Department of Surgery University of Cambridge Cambridge UK; ^8^ School of Health and Related Research University of Sheffield Sheffield UK

**Keywords:** acceptability, cancer, communication, community jury, population screening, risk stratification

## Abstract

**Introduction:**

Using risk stratification to determine eligibility for cancer screening is likely to improve the efficiency of screening programmes by targeting resources towards those most likely to benefit. We aimed to explore the implications of this approach from a societal perspective by understanding public views on the most acceptable stratification strategies.

**Methods:**

We conducted three online community juries with 9 or 10 participants in each. Participants were purposefully sampled by age (40–79 years), sex, ethnicity, social grade and English region. On the first day, participants were informed of the potential benefits and harms of cancer screening and the implications of different ways of introducing stratification using scenarios based on phenotypic and genetic risk scores. On the second day, participants deliberated to reach a verdict on the research question, ‘Which approach(es) to inviting people to screening are acceptable, and under what circumstances?’ Deliberations and feedback were recorded and analysed using thematic analysis.

**Results:**

Across the juries, the principle of risk stratification was generally considered to be an acceptable approach for determining eligibility for screening. Disregarding increasing capacity, the participants considered it to enable efficient resource allocation to high‐risk individuals and could see how it might help to save lives. However, there were concerns regarding fair implementation, particularly how the risk assessment would be performed at scale and how people at low risk would be managed. Some favoured using the most accurate risk prediction model whereas others thought that certain risk factors should be prioritized (particularly factors considered as non‐modifiable and relatively stable, such as genetics and family history). Transparently justifying the programme and public education about cancer risk emerged as important contributors to acceptability.

**Conclusion:**

Using risk stratification to determine eligibility for cancer screening was acceptable to informed members of the public, particularly if it included risk factors they considered fair and when communicated transparently.

**Patient or Public Contribution:**

Two patient and public involvement representatives were involved throughout this study. They were not involved in synthesizing the results but contributed to producing study materials, co‐facilitated the community juries and commented on the interpretation of the findings and final report.

## 1 INTRODUCTION

Many countries offer population‐wide cancer screening programmes, where asymptomatic individuals are offered screening for specific cancers or precancerous changes. Such programmes are only recommended if they fulfil a set of criteria, including evidence that they reduce mortality or morbidity, that the overall benefit outweighs any harms, and that they are acceptable to the public.^1^, ^2^ Most current programmes use age and/or sex to determine eligibility, whereby everyone within the specified age and/or sex bracket is invited. For example, in England all individuals over 60 years are eligible for bowel cancer screening and all women over 50 years are eligible for breast cancer screening.^3^ In recent years, using two or more individual‐level risk factors in combination for risk stratification to determine eligibility for screening has been proposed. This is because risk factors for cancer are not equally distributed across the population and the net benefit of screening is greater among subgroups of the population at higher cancer risk than subgroups at lower risk.^4^, ^5^

Risk stratification has the potential to improve the efficiency of screening programmes by better targeting screening to the population most likely to benefit.^6^, ^7^, ^8^ Thirty‐eight percent of all cancers are attributable to known modifiable risk factors, such as tobacco smoking and body mass index (BMI),^9^ and up to 10% to family history of genetic risk factors.^10^ Potential risk‐stratified approaches could therefore use risk models based on phenotypic and/or genetic risk factors, with people identified at higher risk invited for screening at a younger age or more frequently than those at lower risk.

The potential benefits of such approaches have been demonstrated empirically for a number of cancers.^6^, ^7^, ^8^ However, for screening programmes to be successful, uptake needs to be high. Any introduction of risk stratification must therefore be acceptable to participants and society.^2^ Existing studies assessing the acceptability of risk‐stratified cancer screening have used interviews, focus groups or surveys, and largely focused on breast, ovarian and prostate cancer (all that are only offered to one sex) and views on changing screening intensity. In these studies, the public is generally positive about being offered their risk of cancer and the idea of risk stratification based on genetic testing and other factors, but they tend to be more resistant to less intensive screening for low‐risk individuals than more intensive screening for those at high risk.^11^, ^12^, ^13^, ^14^ Screening programmes have also been shown to have significant symbolic value once they are socially embedded, with invitations reflecting the value society places on individuals.^15^

Additionally, while it is common and acceptable for many aspects of society to be restricted by age or, occasionally, sex, using other factors to determine eligibility for screening raises novel ethical challenges. For example, use of phenotypic risk prediction models may raise questions about the possibility of informed choice to screening; use of genetic markers may reinforce problematic forms of genetic determinism; and, more generally, use of both modifiable and unmodifiable risk factors may raise concerns about fairness and equity, particularly when these factors intersect with other socially salient categories, such as race, disability or social class. Consequently, each element of a stratified screening programme has important implications for individuals and society as a whole, and assessing how the public views these programmes is essential for their success.^16^, ^17^, ^18^

While traditional approaches to seeking public opinions (interviews, focus groups and surveys) enable exploration of the public's intuitive or immediate reactions to problems, deliberative democratic methods, such as community juries, are designed to allow participants to first be informed, and then to discuss, reflect and clarify their own views on a topic. Participants are also explicitly encouraged to think beyond their own interests to consider the collective societal perspective.^19^, ^20^ Deliberative democratic methods are, therefore, particularly valuable for complex, potentially ethically challenging topics, such as cancer screening, when evidence and values are important and people need time to understand and consider relevant issues.^15^, ^21^, ^22^

In this study, we used a deliberative democratic method to explore the social and ethical implications of different ways of using risk stratification to determine eligibility for cancer screening programmes. To inform the National Screening Committee's requirement of public acceptability,^1^, ^2^ we particularly sought to elicit views on the most publicly acceptable stratification strategies and how best to communicate information about them to the wider population.

## 2 MATERIALS AND METHODS

### 2.1 Study design

We conducted three community juries, which we report following the ‘CJCheck Framework’.^23^

### 2.2 Research team

The research team consisted of 12 researchers from diverse backgrounds (including academic clinicians, public health researchers, a public health policy analyst and a medical student) with interests in risk‐stratified cancer screening and/or community jury methods, plus two patient and public involvement (PPI) representatives. The research team met four times over the duration of the project. A subgroup of three researchers plus the PPI representatives designed the protocol, procedures and participant documents with agreement from the wider team. Four of the other researchers presented information to the juries on their area of expertize.

### 2.3 Participants

A market recruitment company recruited participants from across England. Individuals registered with iPoint Research Ltd. were contacted by telephone and asked to confirm demographic and eligibility details and availability for the study. They were purposefully sampled by age, sex, ethnicity, social grade and geographic region. Eligible participants were aged 40–79 years to reflect the age range of individuals currently invited to cancer screening programmes and those below that age who might be invited earlier if identified to be at higher than average population risk. We excluded individuals with expertize in healthcare or a personal history of cancer to reduce the effect personal experiences would have on the discussion.

The recruitment company then allocated available participants to juries so that a range of characteristics were represented in each. They contacted them before the jury to provide organizational details, obtain informed consent, run technology checks (e.g., basic ability to use videoconferencing software) and then provided further study information. The recruitment company reimbursed the participants at their recommended rate.

### 2.4 Procedure

We conducted the community juries using Zoom videoconferencing software (Zoom Video Communications) in April/May 2021. Each were held on two consecutive weekday mornings. The protocol was designed for an online study from the outset because face‐to‐face data collection was not possible at that time due to the coronavirus pandemic.

Before the jury, participants were emailed an information pack containing an introductory letter, timetable, videoconferencing guide, presenters' biographies (available from doi:10.17863/CAM.78681), a summary of a recent survey about introducing risk stratification into cancer screening^24^ and a copy of the presentation slides. They also completed an online questionnaire adapted from a previous study to provide demographic information and their initial, individual attitudes to cancer screening.^24^, ^25^ This included 15 questions concerning how reasonable it seemed or how comfortable they were with experts using age and sex, phenotypic or genetic risk scores to decide when to start screening on a six‐point Likert scale (File S1).

The participants were informed on the first day of each jury while the second day was designed to understand their views (Table 1). Participants were encouraged not to consider specific types of cancer, and to take a population‐based perspective, acknowledging that they would have individual stories about cancer and/or screening. Based on known cancer risk factors and the risk stratification literature,^6^, ^9^, ^10^ suggested approaches to risk stratification included using a phenotypic risk score (that could include risk factors such as age, sex, BMI, smoking, ethnicity, family history and lifestyle) and a genetic risk score. The expert videos (Table 2) were prerecorded and shared on the facilitator's screen. Experts joined the video call after their presentation and were questioned by the participants directly.

**Table 1:** Schedule of events.

Time	Session
Day 1	
From 8.45 AM	Individual welcome and final technology check
9.00 AM	Welcome and introduction (including opportunities to introduce themselves, context, research questions and plan for the community jury)
9.40 AM	Expert video 1—What is screening and what are the potential benefits and harms
10.00 AM	Reflections and Q&A with expert
10.15 AM	*Break*
10.30 AM	Expert video 2—Ethical considerations around screening and determining eligibility
10.50AM	Reflections and Q&A with expert
11.05 AM	Expert video 3—How is eligibility for screening currently determined and what is risk stratification
11.25 AM	Reflections and Q&A with expert
11.40 AM	*Break*
11.55 AM	Expert video 4—The potential effects of introducing risk stratification
12.15 PM	Reflections and Q&A with expert
12.30 PM	Summary of the day
12.45 PM	End
Day 2	
9.00 AM	Check‐in, plan for the day and reflections on the previous day
9.30 AM	Contact experts with outstanding questions
10.00 AM	*Break*
10.10 AM	Jury deliberation, Part 1 (facilitated discussion)
11.10 AM	*Break*
11.25 AM^a^	Jury deliberation, Part 2 (unfacilitated deliberation)
12.25 PM^a^	Present recommendations to screening committee representative (senior author)
12.45 PM	Wrap up and completion of questionnaires
13.00 PM	End

Abbreviation: Q&A, question and answer.

^a^ Timing as required to complete discussions.

**Table 2:** Overview of the expert video presentations.

Title	Presenter	Content of presentation
1. What is screening and what are the potential benefits and harms	General Practitioner and Professor of General Practice	Definition of screening Potential benefits of screening (including prevention, earlier effective treatment and reassurance) Potential harms of screening (including overdiagnosis, overtreatment and anxiety) Most individuals do not derive significant personal benefits from screening
2. Ethical considerations around screening and determining eligibility	Associate Professor of Philosophy of Public Health	Introduction of core principles in medical ethics (do good, do not do harm, treat people fairly and respect choices) How these principles apply to screening The need to balance them when they conflict
3. How is eligibility for screening currently determined and what is risk stratification	Senior Policy Analyst	Current cancer screening programmes invite people of certain ages In stratified screening, the invitation to screening is based on estimated risk Risk can be determined using personal factors including age, sex, BMI, diet and exercise, genetics and so forth
4. The potential effects of introducing risk stratification	Researcher in Primary Care Research	Described a series of scenarios of different strategies for inviting people to screening for a common and uncommon cancer Data were based on a population of 100,000 people aged 40–70 years, modelled on the UK Biobank cohort Reported how outcomes (including number screened and true/false positives) might be different for men and women and older and younger people

Abbreviation: BMI, body mass index.

On the second day, participants engaged in a facilitated discussion followed by unfacilitated deliberation to reach a verdict on our research question (Table 3). Questions for deliberation were shared using Zoom's chat function. At this time, the researchers turned off their videos and microphones but could be contacted using the chat function. Once the participants had reached a verdict, the spokesperson explained this to the senior author, who acted as a representative of a hypothetical screening committee.

**Table 3:** Questions presented to the jury for unfacilitated deliberation.

Question
Main question
Which approach(es) to inviting people to screening are acceptable, and under what circumstances? For example, (1) inviting people when they get to a certain age; (2) use a risk score based on some characteristics (these could include family history, BMI, smoking, ethnicity, socioeconomic characteristics); (3) use genetics.
Follow‐up questions.
Are there any conditions for your selected approach(es)?
What was the most important thing that you heard over the past 2 days that made you come up with your decision?
Anything else you'd like to tell the screening committee?

Abbreviation: BMI, body mass index.

All sessions, excluding the private deliberation, were facilitated by R. D., cofacilitated by R. B. or a PPI representative, and observed by J. U. S. As is usual practice for community juries, all facilitators remained impartial to enable participants to engage in discussion with each other and not be influenced by any prior views of the facilitators. For example, while the facilitators would provide clarification on topics already discussed by the experts, they contacted the experts if additional questions arose rather than providing their own interpretation.

All participant contributions (question and answers [Q&As], discussions, deliberations and feedback) were recorded using Zoom, and participants were reminded that they could see the record symbol on their screen while the recording was in progress.

Finally, the participants completed a second questionnaire to determine whether their individual views on cancer screening had changed during the study and collect their reflections on the process (File S2).

### 2.5 Analysis

An external company transcribed the jury recordings. We analysed them using thematic analysis.^26^ We familiarized ourselves with the data by reviewing video recordings and making notes on the transcripts. Initial coding of surface and implicit meanings was performed using NVivo 12 (QSR International) before collation into potential themes containing unifying concepts. This was an iterative process where codes and themes were revised and refined throughout. Coding was led by R. D. and provisional themes were developed through discussion with the other authors who had attended the juries and/or read the transcripts, before being presented to the entire research team for further discussion and definition. In this paper, we focused on the deliberations and feedback on Day 2 to understand the acceptability of risk stratification to informed participants.

Quantitative data from the pre‐ and postjury questionnaires were analysed using descriptive statistics (Wilcoxon signed‐rank and *χ* ^2^ tests) using Stata 15. To enable us to compare the acceptability of the different approaches to determining eligibility for screening, we generated a single measure of acceptability by calculating the mean of how reasonable participants considered each approach and how comfortable participants were with it.^24^

## 3 RESULTS

### 3.1 Participants

Twenty‐nine participants attended the community juries (9 in Jury 1; 10 in Juries 2 and 3). As reported in Table 4, they varied across a range of characteristics, such as age 40–79 years and household social grades B–D. All participants attended both days of their jury.

**Table 4:** Participant demographics.

	Jury 1 (*n* = 9)	Jury 2 (*n* = 10)	Jury 3 (*n* = 10)	All (*n* = 29)
Sex				
Female	4 (44)	7 (70)	6 (60)	17 (59)
Male	5 (56)	3 (30)	4 (40)	12 (41)
Age category (years)				
40–49	3 (33)	3 (30)	3 (30)	9 (31)
50–59	4 (44)	3 (30)	4 (40)	11 (38)
60–69	1 (11)	3 (30)	2 (20)	6 (21)
70–79	1 (11)	1 (10)	1 (10)	3 (10)
UK region				
London and the South East	4 (44)	5 (50)	6 (60)	15 (52)
North West	3 (33)	3 (30)	0	6 (21)
South West	0	0	1 (10)	1 (3)
West Midlands	2 (22)	2 (20)	0	4 (14)
Yorkshire and the Humber	0	0	3 (30)	3 (10)
Ethnicity				
Asian or Asian British	2 (22)	0	2 (20)	4 (14)
Black or African or Caribbean or Black British	1 (11)	2 (20)	0	3 (10)
Mixed/multiple ethnic group or other	0	1 (10)	1 (10)	2 (7)
White	6 (67)	7 (70)	7 (70)	20 (69)
Education level				
Completed A levels or equivalent, or below	3 (33)	2 (20)	2 (20)	7 (24)
Completed further education but not a degree	3 (33)	2 (20)	2 (20)	7 (24)
Completed a Bachelor's degree, Master's degree or PhD	3 (33)	6 (60)	6 (60)	15 (52)
Social grade^a^				
B (middle–middle class)	3 (33)	4 (40)	3 (30)	10 (34)
C1 (lower middle class)	5 (56)	5 (50)	5 (50)	15 (52)
C2 or D (skilled working class or working class)	1 (11)	1 (10)	2 (20)	4 (14)
BMI category				
Optimal	7 (78)	3 (30)	5 (50)	15 (52)
Overweight	1 (11)	2 (20)	1 (10)	4 (14)
Obese	1 (11)	5 (50)	4 (40)	10 (34)
Smoking status				
Never smoked	4 (44)	8 (80)	3 (30)	15 (52)
Used to smoke	3 (33)	2 (20)	6 (60)	11 (38)
Smoke up to 20 cigarettes per day	2 (22)	0	1 (10)	3 (10)
Self‐rated general health				
Excellent	0	1 (10)	1 (10)	2 (7)
Very good	5 (56)	6 (60)	3 (30)	14 (48)
Good	4 (44)	1 (10)	3 (30)	8 (28)
Fair	0	1 (10)	2 (20)	3 (10)
Poor	0	1 (10)	1 (10)	2 (7)
Family history of cancer				
Yes	6 (67)	5 (50)	2 (20)	15 (52)
No	3 (33)	5 (50)	7 (70)	13 (45)
Don't know/prefer not to say	0	0	1 (10)	1 (3)
Screening history^b^				
Previously invited to screening	6 (67)	9 (90)	7 (70)	22 (76)
Took up the invitation to screening	4 (67)	9 (100)	7 (100)	20 (91)

*Note*: Number of participants (percentage).

Abbreviation: BMI, body mass index.

^a^ Social grade based on the occupation of the chief income earner according to the National Readership Survey.^27^

^b^ Considering abdominal aortic aneurysm screening (men aged over 65 years), bowel cancer screening (men and women aged 60–74 years), breast cancer screening (women aged 50–70 years) and cervical cancer screening (women aged 25–64 years).

### 3.2 Baseline cancer beliefs and views on risk stratification (individual questionnaires)

The participants expressed a range of beliefs about cancer before the juries (Figure S1). Many were positive about treatment, with the majority thinking that cancer could often be cured (*n* = 21, 72%) and all agreeing that going to the doctor after noticing a symptom of cancer could increase the chance of survival (*n* = 29, 100%).

**Figure 1:**
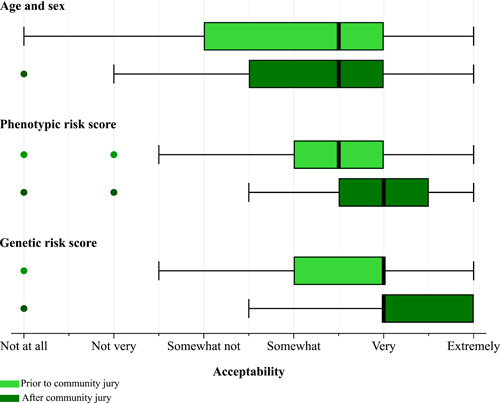
Box and Whisker plots showing how acceptable participants considered using age and sex, a phenotypic risk score, or a genetic risk score to determine eligibility for cancer screening before and after the juries. *Acceptability was based on participants' responses to the questions* ‘How reasonable does it seem to you that experts recommend using [risk factors/score] to decide when to start screening?’ *and* ‘How comfortable are you with experts using [risk factors/score] to decide when you should start screening for this cancer?’ *collected in the pre‐ and poststudy questionnaires on a six‐point Likert scale from* ‘not at all’ *to* ‘extremely’ *reasonable/comfortable*.

Following brief information presented in the questionnaire, incorporating risk stratification within screening programmes based on age and sex or phenotypic risk tended to be well‐accepted, and using genetic risk was slightly more acceptable in comparison (summarized in Figure 1; reported separately in Figure S2A–C). According to the summary measure, 17 participants (59%) found age and sex, 19 (66%) found phenotypic risk scores and 21 (72%) found genetic risk scores at least somewhat acceptable to determine eligibility. There were no statistically significant differences according to age, sex or social grade. However, they were less comfortable with waiting to start screening if they were found to be low risk, with only eight (38%) and six (29%) participants being very or extremely comfortable with this for phenotypic and genetic risk scores, respectively.

### 3.3 Verdict: Acceptability of risk stratification

Across the three juries, the principle of using risk stratification to inform eligibility for cancer screening was generally considered to be acceptable. There was unanimous agreement within Juries 1 and 2 and, although they did not reach a consensus, 6 out of the 10 participants in Jury 3 supported some form of risk stratification. Table 5 reports the spokesperson's conclusion for each jury.

**Table 5:** Juries' feedback on the main research question (which approach[es] to inviting people to screening are acceptable).

	Verdict
Jury 1	*P8: Everybody agreed that screening was essential, but that the more targeted it could be, the better. And that on its own, none of the four elements that are in the first bullet‐point work independently, that it needed to be a combination of all four of them, with the caveat that gender‐based screening, unless it's cancer‐specific, is not acceptable*.^a^
Jury 2	*P17: I think we were comfortable with the various strata to be used in the collation and so forth but I think we felt that family history, genetics, ethnicity were significantly more important than age particularly… it would've been better to concentrate more on the genetic make‐up of people and their family history rather than age alone and to make it more targeted from that point of view*.
Jury 3	*P27: There seemed to be sort of a few camps. Four of us wanted to keep it as it is, the screening process, so just by age and sex. And then there was a second camp which wanted to use the complex risk score but taking some of those factors out, specifically lifestyle factors. So two wanted a complete complex risk score with everything that that involved. Three of us wanted a complex risk score without lifestyle factors, so BMI and smoking but everything else. And one of us wanted a complex risk score without BMI and no family history involved*.

Abbreviation: BMI, body mass index.

^a^ Age, sex, lifestyle characteristics and genetics.

### 3.4 Reasons to accept risk stratification

Numerous reasons were given in favour of using risk stratification within screening criteria. Key concepts centred on increasing efficiency through screening those who will benefit most.

#### 3.4.1 To better identify the people who will benefit most

Risk stratification was understood to be a better strategy for identifying people with cancer than using age alone. Participants could appreciate that people at higher risk would be likely to benefit most from screening and so incorporating additional risk factors to identify those at higher risk seemed sensible (Table 6, Quote 1/2).

**Table 6:** Participants' reasons to accept risk stratification.

Theme	Quotation number	Illustrative quotations
1. To better identify the people who will benefit most	*Q1*	*P12: …using these different factors now which target people who are going to be much more likely to have that cancer rather than just based on their age will hopefully treat more people so that there'll be less people dying and getting into the later stages of cancer because their risks have been identified earlier on*. (Jury 2, unfacilitated deliberation)
	*Q2*	*P13: Obviously that's a lot of work but I think it would actually then mean that the people being screened are the people more likely to have the cancers*. (Jury 2, feedback session)
3. To improve efficiency and better manage resources	*Q3*	*P10: But I don't want the NHS to waste money screening people that are low risk*. (Jury 1, facilitated discussion)
	*Q4*	*P13: You're testing so many people who you think are such low risk that you're actually going to be wasting money*. (Jury 2, unfacilitated deliberation)
5. To increase awareness of cancer risk	*Q5*	*P3: I personally think there should be something on either gov.uk or NHS website that actually lets individuals work out their own risk score, because… what I'm hoping is that it might drive a bit of change in some people… might be very wrong, but it would work for me*. (Jury 1, feedback session)
6. To reduce false‐positive results	*Q6*	*P5: I agree with the lifestyle and the risk score based on their characteristics, and genetics. Those are the things I would be in favour of, and that's because of the statistics that – what horrified me, when I heard that there's such a low positive – false positives*. (Jury 1, unfacilitated deliberation)

Many participants were convinced that there was scientific evidence to support these principles. Consequently, they felt that policy makers should ‘look at the research’ (P15, J2 [Jury 2], unfacilitated [deliberation]) or ‘follow the science’ (P20, J2, facilitated [discussion]) and as such supported risk stratification.

#### 3.4.2 To improve efficiency and better manage resources

Almost all participants strongly supported cancer screening before and throughout the juries. Most participants therefore held the view that more widespread eligibility, specifically screening younger ages, would be ideal. However, in the context of limited resources, the potential for risk stratification to improve efficiency was judged to be important. Some expected that early diagnosis would be cheaper than treating later‐stage cancers so there would be more resources available overall. Others considered that targeting invitations could reduce overall costs by decreasing the total number of people screened to prevent or detect the same number of cancers. A few participants anticipated that it could also enable people with the highest risks to be offered screening at younger ages than current resources permit.

Some participants took this further by considering that not implementing risk stratification was, ultimately, an unacceptable use of resources (Table 6, Q3/4).

#### 3.4.3 To increase awareness of cancer risk

A possible benefit anticipated by the participants themselves was that, alongside education programmes, risk stratification might increase public awareness of how lifestyle choices affect cancer risk, which could motivate behaviour change (Table 6, Q5).

#### 3.4.4 To reduce false positives results

In addition, Jury 1 reflected back on what the experts had presented—that risk stratification was an opportunity to reduce the proportion of false‐positive results and therefore improve the ratio of cases detected to people screened by the programme (Table 6, Q6). Although they rarely associated this with harms of screening other than anxiety, misclassification resulting from screening had been a major concern on the first day of this jury.

### 3.5 Concerns about the fairness of risk stratification

The main issues raised about risk stratification in all juries were ethical concerns, particularly centred on the concept of fairness. For some, these concerns were outweighed by their confidence in the benefits, while they led others to either not accept risk stratification at all or only under some circumstances.

The four participants who elected for screening eligibility to remain age and sex‐based tended to strongly support much more extensive screening, despite the context of limited resources and the effect on the balance between benefits and harms. They were strongly influenced by individual experiences and pre‐existing views, such as ‘A lot of the younger ladies are getting breast cancer, I think, earlier so they definitely need screening, don't they, before the older ones do. That's my opinion. Breast screening needs to go on to, say, from the 30 s onwards…’ (P21, J3, facilitated).

#### 3.5.1 Missing people from the risk assessment

One concern was that some people would miss out on screening because they were not risk‐assessed, such as people who were not registered with a General Practitioner (GP) or whose medical records were out of date. This was seen as a barrier to an inclusive and implementable programme (Table 7, Q1).

**Table 7:** Participants' concerns about the fairness of risk stratification.

Theme	Quotation number	Illustrative quotations
1. Missing people from the risk assessment	*Q1*	*P17: If people don't go to or don't volunteer that information then that whole process is going to become a lot harder anyway*. (Jury 2, feedback session)
2. Risk factors included in the model	*Q2*	*P16: …because the last thing you want to do is to have somebody who was really at risk of catching cancer and only because they didn't know about their mother's history or their father's history they're not invited until they're 55*. (Jury 2, facilitated discussion)
	*Q3*	*P17: I just thought that family history and genetics, you don't have a lot of choice and I don't want to bring in choice too much because some people say it's lifestyle choice and it isn't*. (Jury 2, unfacilitated deliberation)
	*Q4*	*P3: …So it brings everything in the picture—* *P6: Yeah, the risk score's covering everything, isn't it?—* *P2: The lifestyle and everything.P8: But who decides on the weighting of those risks?* *P3: The big professors that came up with the plan*. *P2: GPs and doctors who have got our information*. (Jury 1, unfacilitated deliberation)
5. Management of people at low risk of cancer	*Q5*	*P5: Except that they will lose out on a lot of people that don't have any of those characteristics but still get cancer*. *P10: I'd say that is the thing that sits most uncomfortably with me, but I do recognise it is those added lifestyle factors that mean that that person is more likely to develop cancer, so we have to screen those people*. (Jury 1, unfacilitated deliberation)
	*Q6*	*P10: I do struggle with that a little bit because it's almost like those that are unhealthy are going to get the screening and those that are healthy aren't*. (Jury 1, facilitated discussion)
	*Q7*	*P29: I feel that people who've tried to lead healthy lives may be excluded unfairly. This is the ‘unfairly' bit where it's not based on, you know, actual… I don't know how to put it but that to me would be unjust*. (Jury 3, unfacilitated deliberation)
8. Adequate resources for screening	*Q8*	*P8: …the more people are being picked up, the greater the risk of a bottleneck further on down the line, which then exacerbates both the emotional impact and potentially the physical consequences of the cancer*. (Jury 1, feedback session)

After weighing up various factors, P24 concluded that ‘basically it's got to come down to age and sex again just to sort of not be biased towards anybody’ (J3, unfacilitated). This participant felt that requiring a risk assessment would compound existing barriers to screening for people of certain ethnic minorities, such as social norms around healthcare‐seeking or stigma, and, hence, health inequity.

#### 3.5.2 Risk factors included in the model

There was considerable discussion about the specific factors that could make up risk models (Table 8). Some participants independently distinguished between nonmodifiable, relatively stable and easy‐to‐measure factors—listing genetics, family history and ethnicity—and lifestyle characteristics. Consequently, there were differing views on how the factors should be used: Jury 1 and some participants in Jury 3 supported an optimized risk prediction model; Jury 2 and other participants in Jury 3 felt some characteristics were inherently more important than others.

**Table 8:** Views on specific risk factors that could be used in risk stratification.

Risk factor	Summary	Illustrative quotations
Age	Age was considered an important and well‐understood cancer risk factor. Everyone who accepted risk stratification considered that age should be included.	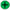 *P12: Because there's obviously all of this study gone into age before what they're doing now and there's a reason why you get invited to have bowel cancer screening at… I think is it 60… and breast cancer screening and prostate screening. So I think age is actually quite an important factor*. (Jury 2, unfacilitated deliberation)
		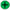 *P23: …it's up to the statisticians, I suppose, to work out where most of the cancers are and at what age they are happening, and then decide on when they're going to start the screening programmes*. (Jury 3, unfacilitated deliberation)
Sex	Particularly early on in the juries, many participants were uncomfortable about using sex in determining eligibility for screening. Later on, although they wanted equal screening for men and women, it was more acceptable when used in combination with other factors and justified biologically.	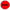 *P5: No, because – okay, prostate cancer, [women] don't have a prostate, so that's logical, but anything else, general cancers, no*. (Jury 1, unfacilitated deliberation)
		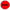 *P16: …what about those people who don't identify in terms of a gender?* (Jury 2, unfacilitated deliberation)
		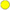 *Researcher: …whether you would consider sex as a risk factor alongside BMI, smoking, other lifestyle factors, genetics, etc, whether you would consider that to be acceptable, or did you discuss that?*
		*P8: We did, and I think that the issue is that it needs to be cancer specific, that if there is evidence that there is a greater prevalence of a particular cancer then that feeds into the risk factors. But if there isn't that evidence then assumptions shouldn't be made that the targeting would be male or female*. (Jury 1, feedback session)
Lifestyle	A key point of discussion was the extent to which lifestyle (such as smoking, diet and physical activity) was within an individual's control. As a result, many who felt that it was a choice did not consider it fair to include lifestyle within risk models, and vice versa. The extent to which they were convinced it was associated with cancer risk also influenced this decision.	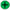 *P10: I'd say that is the thing that sits most uncomfortably with me, but I do recognise it is those added lifestyle factors that mean that that person is more likely to develop cancer, so we have to screen those people*. (Jury 1, unfacilitated deliberation)
		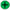 *P12: Some lifestyle choices are actually big factors as well*. (Jury 2, facilitated discussion)
		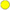 *P17: With lifestyle I'm a little bit thinking both ways because on the one hand you could say that's a lifestyle choice and people that abuse themselves with drugs, alcohol, smoking… Do we classify them as bad people or are those habits they have brought on by the environments they live in and depravity and so forth?* (Jury 2, facilitated discussion)
		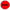 *P14: It sounds a bit cruel, this, but should we be wasting our time on them type of people that aren't interested when you've got people that might want to know? I know it's very controversial*. (Jury 2, facilitated discussion)
BMI	Closely linked to lifestyle, individuals had divergent perspectives on whether BMI/being overweight was the result of individual choices or a result of circumstances and opportunities outside the control of individuals, and whether it was associated with cancer risk or not, despite discussion with the experts. Consequently, many different views on including it in risk models were presented.	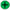 *P12: We all know that if you are obese or morbidly obese it puts you at risk for lots of different diseases, cancer being one of them… So I think it's a bit dismissive to say that lifestyle choices are people's choices because they're not always and sometimes people need a lot of help with things. But I think for me definitely weight is a big thing from what the GPs said*. (Jury 1, facilitated discussion)
		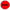 *P23: Why would you turn around and use an athlete that's got a BMI of, say, 40? It's completely inaccurate, isn't it?*
		*P24: Yeah, I think the BMI is pretty flawed, to be honest. You know, I definitely don't agree that you should go on BMI because there's lots of variables on that*. (Jury 3, facilitated discussion)
Geography and environment	Jury 1 suggested including locality to try to address observed health inequalities. Along similar lines, Jury 2 also wanted to include it as an indicator of pollution, although difficulties in measuring the data were raised. It did not come up in Jury 3.	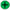 *P8: The one final thing that we had a bullet‐point about was other risk factors may be taken into consideration, such as locality, knowing that some areas, even down to quite small districts, have a higher propensity of certain cancers, so should we be looking at, you know, one part of the country, but even maybe one part of the local authority as opposed to another*. (Jury 1, feedback session)
		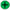 *P20: So, you know, how many people with a certain cancer after a certain age, have a look at where they live. So if you live in an urban environment is there a greater propensity to have a cancer or have an illness or be less well than in an environment where you've less population density, more green, fewer cars, that tends to suggest more affluence, but that would be self‐evident from your address so that can be screened out*. (Jury 2, unfacilitated deliberation)
Ethnicity	Juries 1 and 2 supported including ethnicity within risk prediction models, as long as it was clearly justified and communicated. They considered it to predict cancer risk and to be closely linked to genetics and family history. Ethnicity was not discussed in Jury 3.	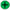 *P17: The other thing that you can't really argue with is ethnicity… it could be certain [ethnic] groups would be more predisposed to certain things. P16 brought up the point about prostate cancer. [Their] ethnic group potentially is more likely to contract that than my ethnic group (overspeaking)—*
		*P13: But in [their] family history that should come up and therefore be tested, if you see what I mean. P12: Yeah, ethnicity is only one of the factors within that whole bulk of factors, isn't it, so it's BMI, smoking, ethnicity, weight, healthy eating, so that's just one of the factors*. (Jury 2, unfacilitated deliberation)
		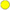 *P11: People could then say, ‘Well I'm being targeted, why am I being targeted because of my ethnicity?’ so that could be quite detrimental*. *Researcher: Yeah. I guess it comes back to the first comment that P17 made about your discussions, that it's really important how this is communicated to people*. *P20: Crucial*. (Jury 2, feedback session)
Family history	Again, family history of cancer was considered to be an undisputable factor, equivalent to ethnicity and genetics, particularly in Jury 2 (although some expected that it might be redundant if genetics were included). Many felt that people with a family history of particular cancer should be able to be screened for it at a young age, which others understood was already current practice. A disadvantage was that some people don't know their family history, or it might be irrelevant, creating possible unfairness in access.	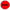 *P16: Yeah, with [family history], you know, there is going to be a particular group what's going to be left out, I think we established that yesterday, those people who have been adopted, because they won't have their family history*. (Jury 2, facilitated discussion)
		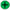 *P24: I think we need to look at the factors that are stable, so it is genetics, it is, you know, looking at family history and those sort of things, that would be more of the appropriate methods*. (Jury 3, facilitated discussion)
		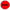 *P23: If you take the ‘60 s’ and ‘70 s’ we had what they call the ‘smog’, you know, the fog, and it wasn't fog, it was basically coal gas, you know, and the dust from coal fires, and that's what was predominantly causing cancers in all ages, so where do you stop with family history, you know? So for me that's just not accurate for today's times*. (Jury 3, unfacilitated deliberation)
		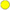 *P29: [P22] said that if someone's already got a family history they're already testing it, if I'm correct in what I heard. Is that correct, P22?* *P22: Yeah. Yeah, because my cousins have got the BRCA gene*. *P29: …. We may actually say, ‘We don't need to do this extra family testing. Because you've got a potential running in the family history, we don't need to do two tests’. The family doesn't need to have this second test because it'll become part of the overall test, so when we say actually it's age, sex, genetics and actually, by definition, family history, because we've already done that and we know it's a tick in the box*. (Jury 3, unfacilitated deliberation)
Genetics	The majority of the participants were very positive about including genetics and seemed to believe that it was a reliable and significant risk predictor that could be measured at a young age. That said, many seemed to believe that all genetic risk was inherited by a few dominant genes. Also, they expressed concerns about collecting genetic information.	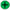 *P3: For me, it's the explanation from Simon today about genetic codes and how it's used, how it's applied. It's created more awareness and led me to like actually accept that as a means or as part of like the risk score approach*. *P1: I fully agree with P3 about the genetic thing. It's opened my eyes that it's not as big and bad and scary as it may be promoted*. (Jury 1, unfacilitated deliberation)
		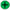 *P17: A show of hands. Who thinks genetics and family history should be the prime factor?[7/10 clearly raise hands]* *P15: I think it should be weighted heavily, yeah… the most significant indicator*. (Jury 2, unfacilitated deliberation)
		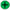 *P29: …that actually gives you the confidence that we're actually getting the right people. So I know the genetics will scare people off but actually by having that as part of the complex testing… And you may say age, sex and genetics but including genetics is actually hard fact. I don't have to tell my GP I drink twenty or whatever, it's there, they take the blood test, it's factual. … All of the [other lifestyle factors], ‘Ooh there's a chance’, but you've got this BRCA gene – don't ask me what it is – but it's bad or there's a possibility that you could get something, that gives us some certainty*. (Jury 3, unfacilitated deliberation)
		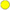 *P22: And obviously the thing about human rights and the ethics connected to genetics, you know, we can't secretly test people to see if they've got cancer genes so how do we do it, ensuring that people are turning up to do those tests?* (Jury 3, feedback session)

Abbreviation: BMI, body mass index.

*Note*: Positive comments (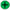), negative comments (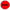), and neutral or mixed comments (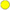) about using the risk factor within risk stratification to determine eligibility for cancer screening.

The participants who favoured a selective approach would exclude certain factors or give them a lower weighting in the risk calculation, overlooking predictive ability. They considered that factors for risk stratification should be constant and objectively measured because they were concerned that data would become inaccurate (e.g., if someone lost weight) and were concerned about missing data (Table 7, Q2). Some also worried that people might lie about or exaggerate self‐reported data, giving them an unfair opportunity to claim social resources.

Focussing on nonmodifiable factors also avoided the dilemma between not favouring people who deliberately made unhealthy choices and that such people are more likely to develop cancer (Table 7, Q3).

In addition, others were unconvinced that some factors could predict cancer risk and therefore rejected including them in risk models. They considered that BMI was a poor measure of weight‐related cancer risk or that family history was ‘a complete waste of time’ because older generations had different lifestyles with different health pressures such as pollution and *‘*a huge amount of stress compared to us’ (P24, J3, facilitated).

The other participants supported stratification that incorporated the best possible estimate of cancer risk. For these individuals, all of the risk factors in Table 8 were considered ‘just as relevant’ (P2, J1, unfacilitated) and should be included as long as there was evidence that they contributed to cancer risk. This was particularly important when considering including sex in these models because ‘if there isn't that evidence then assumptions shouldn't be made that the targeting would be male or female’ (P8, J1, feedback [session]). They were therefore eager for scientists and medical experts to determine the risk prediction model based on the best scientific evidence (Table 7, Q4).

#### 3.5.3 Management of people at low risk of cancer

A further aspect of fairness that arose across all three juries was participants' struggle with the implications of risk stratification for those at low risk of cancer, particularly as many knew ‘fit’ people who had become ill.

Many concluded that it was unfortunate for such people to miss screening or be invited later, but most of them were unlikely to benefit therefore risk stratification was necessary to gain the benefits described above (Table 7, Q5).

In a Q&A session in Jury 3, one of the experts explained that it was unlikely that people identified as low cancer risk would never be invited for screening, just that it would be at an older age than people at a higher risk. This element was important for members of Jury 3 to accept risk stratification. They anticipated that ‘there may be some initial discrimination but eventually when you reach a certain age or you're eligible to have a genetics test or whatever you are going to get screening at some point’ (P27, J3, facilitated). In contrast, others considered that it was unjust or unfair for people who strive to be healthy not to be offered this service (Table 7, Q6/7).

Of note, none of the juries considered the avoidance of some harms of screening (introduced on Day 1) in people at low risk, such as screening anxiety or potentially unnecessary follow‐up, to be a significant benefit of risk stratification.

#### 3.5.4 Adequate resources for screening

Collectively, Jury 1 was concerned that incorporating risk stratification would lead to more people needing follow‐up and treatment, therefore they would need assurance that sufficient resources were available to meet these higher requirements before supporting it (Table 7, Q8). Additionally, investment in developing more accurate screening tests was important.

### 3.6 Conditions for introducing risk stratification: Transparency and public education

Finally, in addition to ensuring the fairness of the programme by addressing the concerns described in Section 3.5, including opportunities for the public to learn about cancer risk was a condition for the implementation of risk stratification to be acceptable in all of the juries. This should include communication about why the factors were included in the risk assessment, and preventative advice linked to those risk factors integral within the screening programme. Furthermore, using simple‐to‐understand language in communication and being transparent about the screening programme as a whole (from risk assessment to diagnostic testing) were key.

### 3.7 Change in cancer beliefs and views on risk stratification (individual questionnaires)

Participants' individual beliefs about cancer tended not to change after the expert presentations and jury discussions (Figure S1), yet they were more convinced that they would want to know if they had cancer (*p* = .033).

Their views on risk stratification either remained similar to baseline or increased in favour of risk stratification, particularly for using genetic risk scores (Figures 1 and S2A–C). Twenty‐three participants (79%) found phenotypic risk scores and 25 (86%) found genetic risk scores at least somewhat acceptable to determine eligibility after the juries (*p* = .038 for the change in the combined measure of acceptability for genetic risk scores). The participants also were more comfortable waiting to start screening if they were found to be at low risk of cancer using genetic risk scores compared to baseline (*p* = .010).

### 3.8 Evaluation

Overall, the participants had positive reflections on the juries (Figure S3). Although a few participants would have appreciated more time to discuss new, complicated concepts, they indicated that they had considered the information provided by the experts and that ethical considerations were key in influencing their views (Tables 9, Q1–3).

**Table 9:** Participants' reflections on the community jury process.

Theme	Quotation number	Illustrative quotations
Evaluation	*Q1*	*P5: As we've been educated and learnt more about the subject, and we've delved into it, and we've listened to others' opinions, that's why we came up with the conclusions that we did*. (Jury 1, feedback session)
	*Q2*	*P1: For me, it was the ethics and what an actual nightmare that must be to decide, because every turning, with the ethics argument, was correct… And it made me look at it a lot differently, you know. It's not just about the medicine*. (Jury 1, unfacilitated deliberation)
	*Q3*	*P24: Very interesting to listen and discuss with different people. Differences in opinions and factors that gave us a different perspective*. (Jury 3, questionnaire)

Importantly, they felt that it had been an interesting experience and had valued sharing their views with others. They also felt that the study was worthwhile for researchers to ‘have a better understanding of the concerns the public have’ (P23, J3, questionnaire).

## 4 DISCUSSION

### 4.1 Principal findings

**Figure 2:**
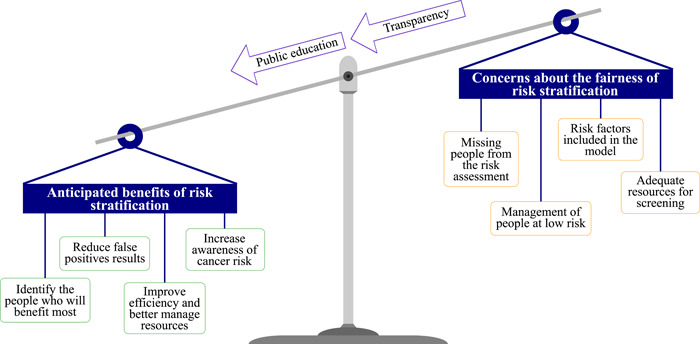
Summary of juries' deliberations on the acceptability of using risk stratification to determine eligibility for cancer screening.

This is the first study to investigate in‐depth societal views on the general concept of using risk stratification to determine eligibility for cancer screening. For the majority of participants in our study, incorporating additional risk factors would be more acceptable than current age‐based strategies, as long as it was explained well. Significant ethical concerns were discussed, including avoiding inadvertent discrimination and not reinforcing existing inequalities when conducting the risk assessment. However, the majority of participants felt that the potential for risk stratification to improve early detection, resource efficiency, cancer awareness and test accuracy outweighed these harms once they were informed (Figure 2). Consequently, communication strategies will be central to future policies that include risk stratification in screening eligibility. They must include transparent explanations of how individual risk would be calculated and used.

### 4.2 Comparison with existing literature

Our participants' positive acceptance of risk stratification is consistent with findings from previous research in less informed participants that focused on specific cancer types.^11^, ^12^, ^13^, ^24^, ^28^, ^29^, ^30^, ^31^, ^32^ The possibility for risk assessment to support behaviour change has also been made by members of the public previously.^24^ Despite being presented with quantitative data on the potential benefits, the scale of the benefits did not appear to be important. While evidence was necessary to support their decision, risk stratification was viewed favourably if there was any benefit. This is consistent with results from a survey about the intention to attend bowel cancer screening, where most people were not influenced by the absolute benefits and harms presented.^33^

In contrast to other studies, we did not observe a focus on the negative implications of a high‐risk classification (such as distress, potential pressure for lifestyle changes or the burden of being high‐risk for multiple cancer types).^28^, ^34^ Reduction in the potential harms of screening for those at low risk was also not a consideration, emphasizing that this may be of greater concern for researchers and clinicians than the public. Instead, our participants focused on the potential for low‐risk individuals to miss out on screening. Such concerns around accepting reduced screening for those at low risk have been reported previously.^13^, ^14^, ^28^, ^30^, ^31^, ^32^, ^35^ Coupled with findings that overdetection in population screening is acceptable to the public and that many are willing to undergo diagnostic testing regardless of cancer risk,^36^, ^37^ this reinforces the relatively low importance placed by the public on the potential harms of screening people at low risk. We were able to observe that among our participants this was a matter of fairness, with many initially seeking *equality* in screening (meaning the equal provision of and access to screening services, according to Sasieni's definition) before considering the implications and becoming more favourable to a more *equitable* risk‐stratified approach (attempting to equalize cancer outcomes).^38^ In agreement with McWilliams et al.,^35^ this highlights the importance of evidence and effective communication in order for risk stratification to be acceptable.

A new finding in this study was the significance participants gave to the effects of risk stratification on the wider healthcare system, specifically the potential to increase the efficiency of the screening programme while also requiring capacity for more diagnostic tests. These aspects may have been particularly salient to our participants because our study took place during the coronavirus pandemic when awareness of the demands on health services might have been higher.^39^, ^40^

Additionally, we were able to gain a more detailed understanding of public views on risk factors that could be included in stratification than in previous studies, particularly concerning lifestyle factors. In an earlier study, we found that a complex risk score (based on age, sex, BMI, smoking status, family history and lifestyle) was more acceptable than a simple one (excluding family history and lifestyle), but were not able to comment on individual risk factors apart from sex alone, which was least acceptable.^24^ Our findings in this study show how important the distinction between modifiable and nonmodifiable risk factors (and whether a change in risk occurred by personal lifestyle choices) was for some participants and the impact that had on acceptability. Our findings add to the growing body of research showing support for genetic risk^11^, ^41^, ^42^: genetics were considered to be accurate, consistent over time and free from complex ethical debates over personal responsibility for health.

### 4.3 Strengths and limitations

The use of community juries is the central strength of this study. By providing information from experts and then encouraging participants to deliberate as a group, we were able to generate data that provide us with an in‐depth understanding of concerns and priorities amongst informed members of the public. We encouraged participants to consider a societal perspective, and therefore they thought about the implications of their recommendations for people both like and unlike themselves (e.g., who were not registered with a GP). Despite the complexity of some of the information, many participants indicated that they made use of it when forming their views. The inclusion of an expert presentation on ethical principles to provide them with a framework upon which to think about the topic was an especially useful and unique aspect.

Although there was evidence that the participants understood many of the complicated concepts we presented, they still appeared to base some of their decisions on misunderstandings. This was often because they were not convinced by the data (e.g., the association between BMI and cancer), or they had preconceived ideas that were raised in unfacilitated deliberations (such as that all genetic cancer risk is attributed to a single high‐penetrance gene).

The online nature of the juries also meant the dynamics of the deliberations were likely different from if they had been held face‐to‐face and may have limited opportunities for questions. However, the postjury evaluation showed that participants had positive reflections on the juries and had felt able to contribute their views. Our use of videoconferencing also meant we were able to include people from diverse backgrounds and different regions of the country. Although people with an interest in cancer screening may still have been more likely to take up the study invitation, this approach enabled a sample that is more diverse overall than is usually possible in qualitative research.

### 4.4 Implications

In accordance with screening programme principles, the programme as a whole should be clinically, socially and ethically acceptable to society and the individuals undergoing screening.^1^, ^2^ Additionally, acceptability is necessary for uptake and therefore overall effectiveness. We found that incorporating risk stratification into cancer screening eligibility meets these criteria. This will be important for developing new population screening programmes or amending existing ones.

We have also highlighted that the implementation of risk stratification must be cohesive and transparent. Members of the public should be involved in developing and testing each element of the programme and the rationale for the approach should be clearly justified. Our findings suggest that reducing potential individual harm to those at low risk is less of a consideration to the public than increasing potential benefits to those at high risk. It will be important to address anticipated positive outcomes for early detection, and clarify that the aim is to focus on those most likely to benefit and not to deny people screening, since everyone could be invited at some point. The context of insufficient resources to screen everyone will also be important to convey. Moreover, information about how and why each of the factors is included in the risk assessment must be easily available and understandable. This is particularly important for risk factors that are, to some extent, influenced by individual choice (such as BMI) and protected characteristics (such as sex and ethnicity), because subgroups of the population may be perceived to be being discriminated against, either positively or negatively. Additional considerations to increase the appeal of risk‐stratified screening include measures to ensure that everyone has the opportunity for risk assessment and that cancer prevention information is provided alongside risk.

## 5 CONCLUSION

We found that informed members of the public supported using risk stratification to determine eligibility for cancer screening programmes. While many benefits of incorporating additional risk factors were salient, the entire programme had to be perceived as fair to be acceptable. As a result, public communication, especially education about cancer risk and comprehensive justification for risk stratification, will be essential components for revising eligibility criteria.

## AUTHOR CONTRIBUTIONS

Juliet Usher‐Smith conceived the idea for the study, which was led by Rebecca Dennison and Juliet Usher‐Smith. All authors and patient and public involvement (PPI) representatives contributed to the design. Rebecca Dennison, Juliet Usher‐Smith and Rachel Boscott or PPI representatives facilitated the community juries; Simon Griffin, Hannah Harrison, Stephen John and Sowmiya Moorthie presented in the community juries as experts. Rebecca Dennison led the analysis, along with Juliet Usher‐Smith, Rae Thomas and Rachel Boscott. All authors discussed the findings and interpretation. Rebecca Dennison wrote the manuscript, to which all authors have given substantial suggestions and feedback, and approved the final version.

## CONFLICTS OF INTEREST

Grant Stewart has received educational grants from Pfizer, AstraZeneca and Intuitive Surgical; consultancy fees from Pfizer, Merck, EUSA Pharma and CMR Surgical; travel expenses from Pfizer and speaker fees from Pfizer. All other authors have no financial disclosures or conflicts of interest with respect to the research, authorship, and/or publication of this article.

## ETHICS STATEMENT

Ethical approval was provided by the University of Cambridge Psychology Research Ethics Committee (PRE.2020.153).

## Supporting information

Supplementary Information

## Data Availability

Pseudo‐anonymized transcripts are available via the University of Cambridge Data Repository: doi:10.17863/CAM.78681. Formal requests for access will be considered via a data‐sharing agreement that indicates the criteria for data access and conditions for research use and will incorporate privacy and confidentiality standards to ensure data security.
