# Supplementary material for: A community jury study exploring the public acceptability of using risk stratification to determine eligibility for cancer screening
Source: Health Expect. 2022 May 8;25(4):1789–806. doi: 10.1111/hex.13522 (PMC9327868; doi:10.1111/hex.13522)
Supplement: Supplementary file 1 — Supplementary Information [file HEX-25--s001.pdf]

A community jury study exploring the public acceptability of using risk stratification to determine eligibility for cancer screening

Supplementary figures and files

Supplementary Figure 1. *Participants' beliefs about cancer before and after the community jury.*

Below are some statements that are sometimes made about cancer. For each of these statements how much do you agree or disagree with them?

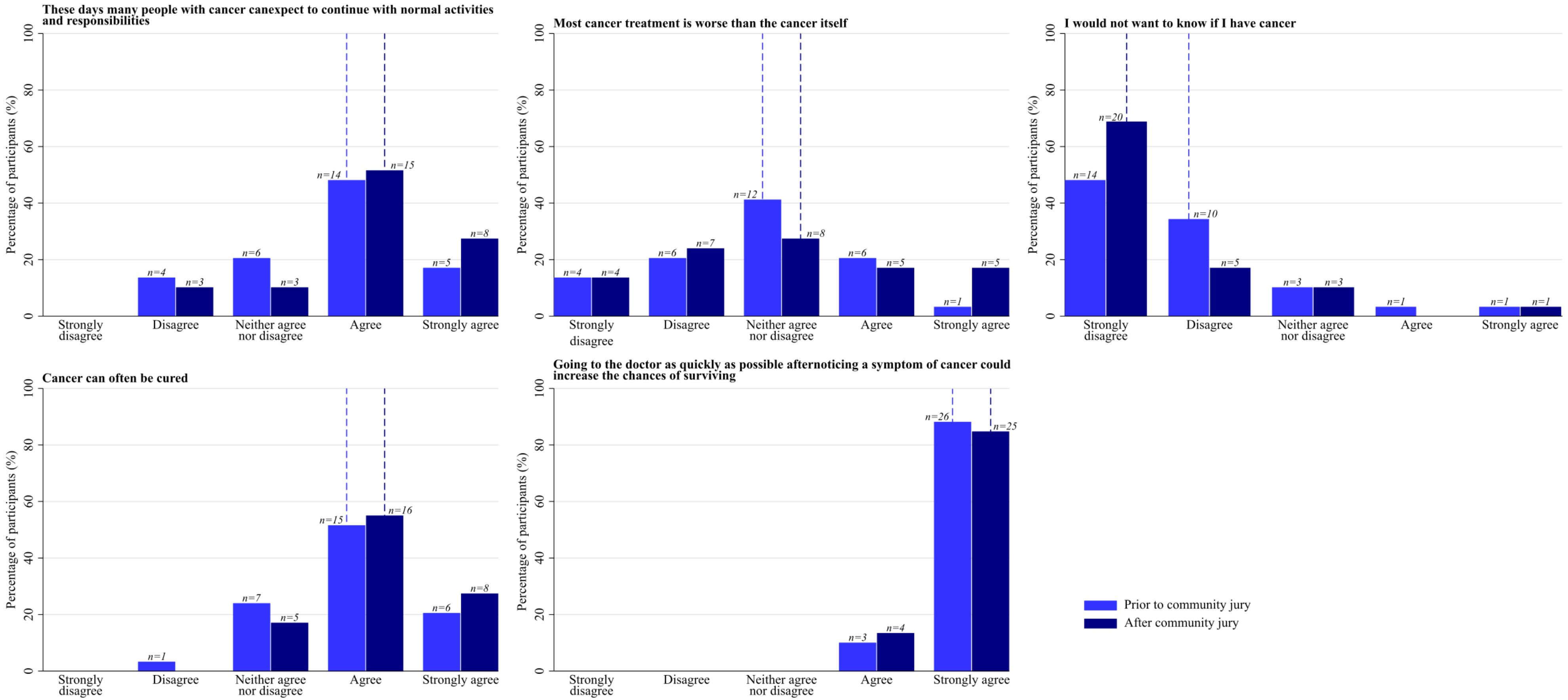

Histograms showing participants' responses. Dotted lines indicate the median.

Supplementary Figure 2. *Participants' views on risk-stratified cancer screening before and after the community jury.*

A. Age and sex

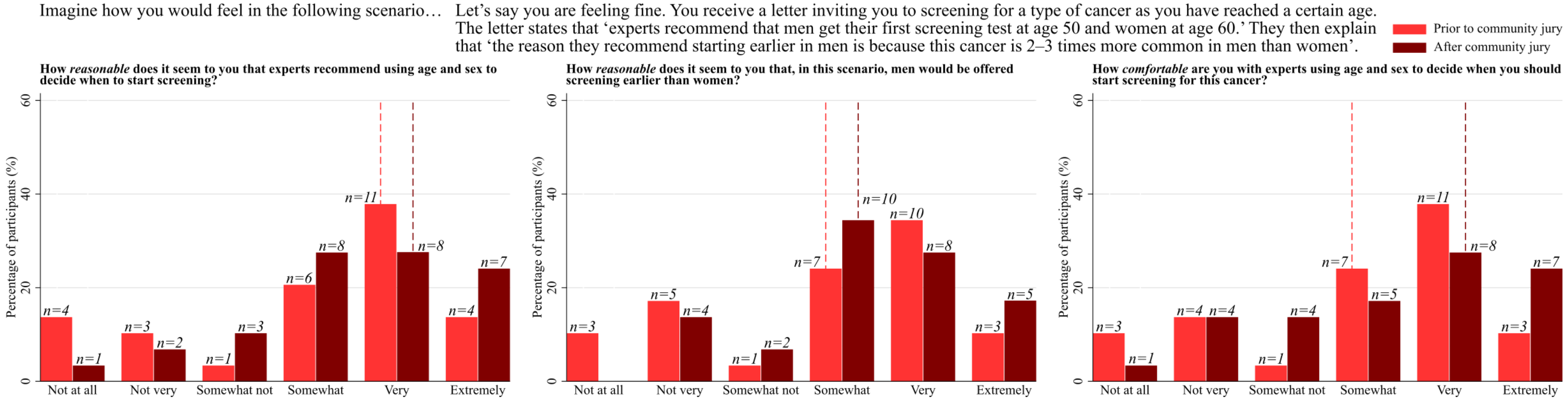

Histograms showing participants' responses. Dotted lines indicate the median.

B. Phenotypic risk score

Now imagine that you receive a different letter inviting you to screening. In that it describes how experts have developed a calculator to estimate how likely an individual is to develop a type of cancer, and to identify at what age you should first have screening for this cancer.

This calculator, which is based on scientific research studies, uses information available from routine GP records, such as your age, sex, BMI (body mass index, a measure that uses your weight and height to work out if your weight is healthy) and smoking status. You are also asked to fill out a questionnaire for the calculator, which asks about your family history of cancer and your lifestyle, including how much physical activity you do and your diet.

Like the screening tests, it is not 100% reliable. Someone who is estimated to be at higher risk based on the calculator is not destined to get this cancer. A low risk estimate also does not mean someone is completely without risk of getting this cancer.

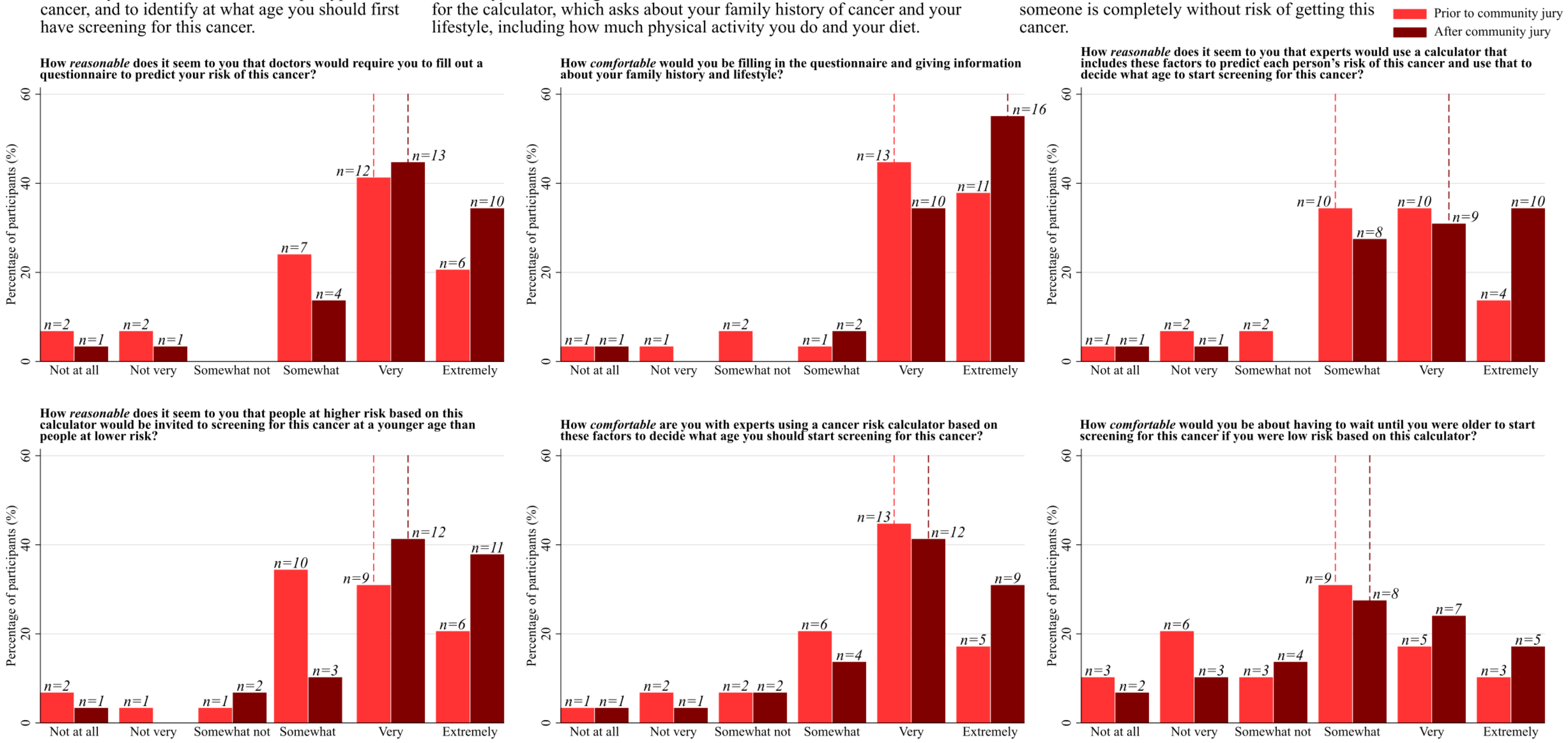

Histograms showing participants' responses. Dotted lines indicate the median.

C. Genetic risk score

Having certain genes can also increase your risk of a type of cancer. Imagine that you are invited to be tested for these high-risk genes by providing a sample of blood or a cheek swab. This information would then be used to calculate how likely you are to develop this cancer and used to decide what age to start screening.

The relationship of these genes with the risk of this cancer is based on scientific research. Like the risk calculators above, the calculator based on genes is not 100% reliable. Someone who is estimated to be at higher risk based on their genes is not destined to get this cancer. A low risk estimate also does not mean someone is completely without risk of getting this cancer.

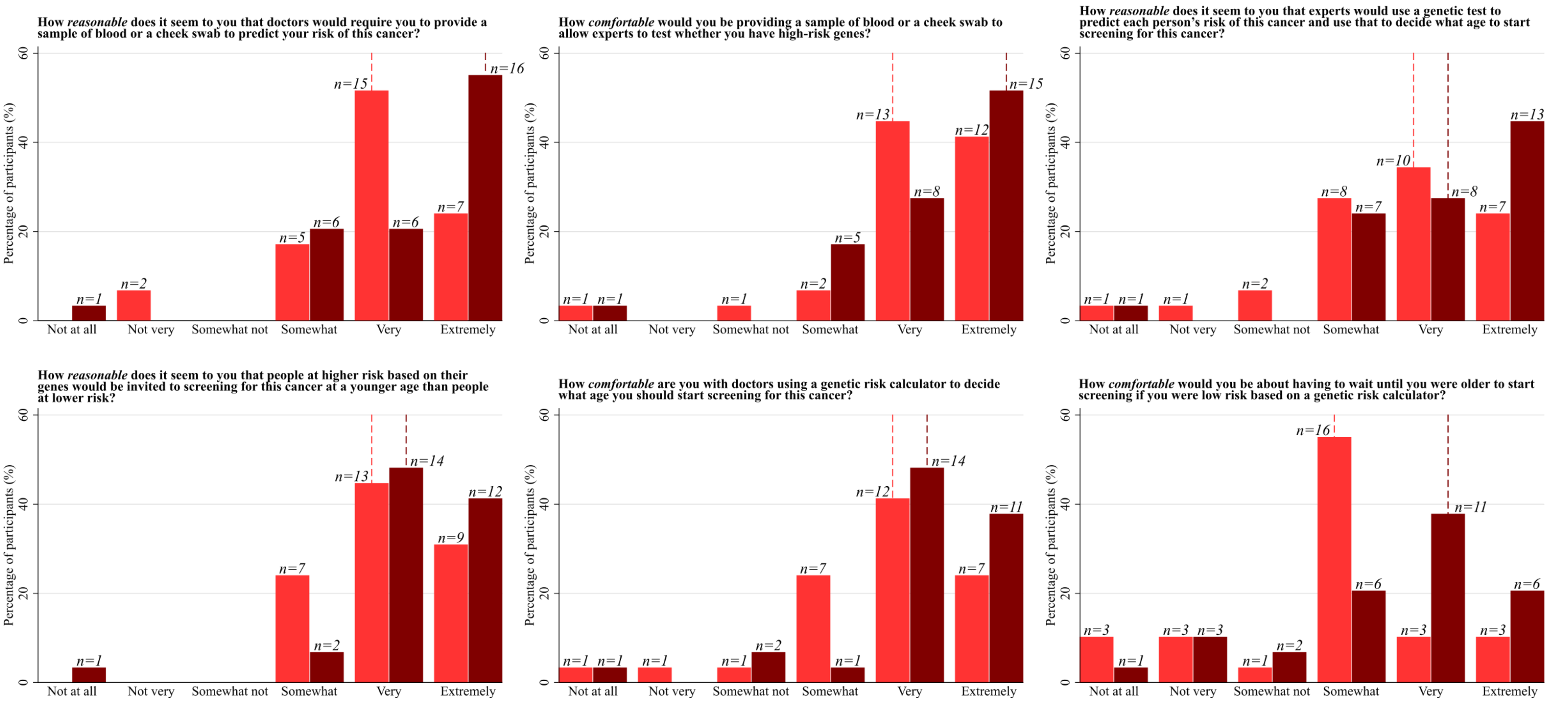

Histograms showing participants' responses. Dotted lines indicate the median.

Supplementary Figure 3. *Participants' feedback on their experience of the community jury.*

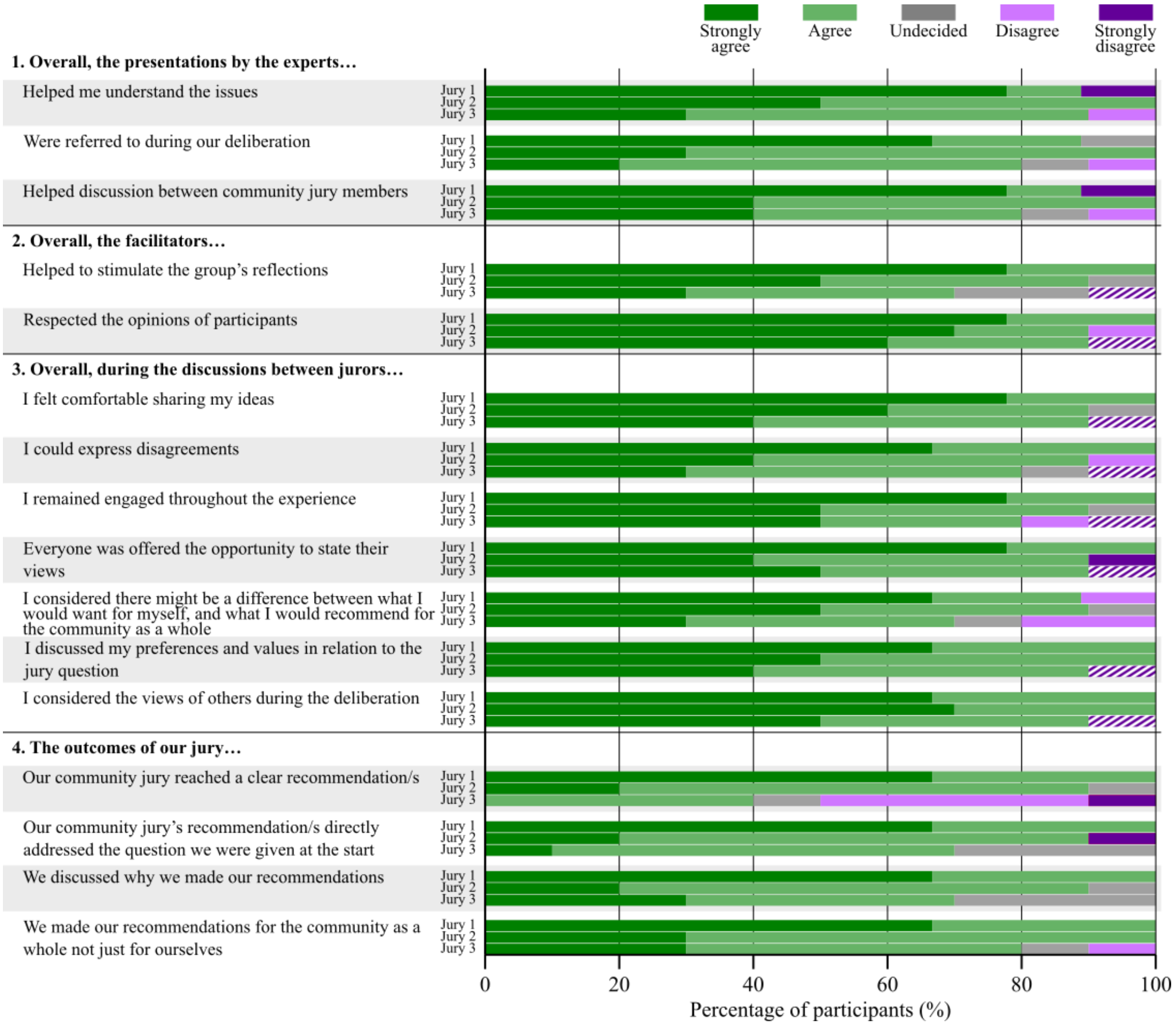

Note: One participant in Jury 3 responded 'strongly disagree' to questions 2 and 3 (shaded with hashed) but provided contradictory free-text comments suggesting that they had positive experiences of the facilitators and deliberations.

Supplementary File 1: *Questionnaire 1 (completed before community jury)*

[This content was delivered online. The participants did not see the headings in grey boxes.]

Thank you for agreeing to complete this questionnaire. Please answer every question. If you are uncertain about how to answer a question, then please select the closest option.

Demographic information

In this first section we would like to ask you a few questions about yourself. These questions allow us to make sure we are including people from a range of different backgrounds and see if different groups of people have different views. We will not be able to identify you from your answers.

- **How old are you?**
  - 40-44 years
  - 45-49 years
  - 50-54 years
  - 55-59 years
  - 60-64 years
  - 65-69 years
  - 70-74 years
  - 75-79 years

- **What is your sex as assigned at birth?**
  Some of the scenarios we will be discussing will consider whether the sex of individuals should influence when they are invited to screening. This would be the sex that individuals are assigned at birth. To help us see if responses differ between people of different sexes, please let us know your sex assigned at birth.
  - Male
  - Female
  - Don't know/prefer not to say

- **What is your ethnic group?**
  Choose one option that best describes your ethnic group or background.
  - White
  - Mixed/multiple ethnic group
  - Asian/Asian British
  - Black/African/Caribbean/Black British
  - Other, please describe ______

- **What is your highest education level?**
  - Finished school at or before the age of fifteen
  - Completed GCSEs, O Levels or equivalent
  - Completed A Levels or equivalent
  - Completed further education but not a degree
  - Completed a Bachelor's degree/Master's degree/PhD
  - Other (please specify) ______

- **Please indicate to which occupational group the Chief Income Earner in your household belongs, or which group fits best.**
  This could be you: the Chief Income Earner is the person in your household with the largest income. If the Chief Income Earner is retired and has a pension please answer for their most recent occupation. If the Chief Income Earner is not in paid employment but has been out of work for less than 6 months, please answer for their most recent occupation.
  - Professional or higher technical work – work that requires at least degree-level qualifications (e.g. doctor, accountant, schoolteacher, university lecturer, social worker, systems analyst)
  - Manager or senior administrator (e.g. company director, finance manager, personnel manager, senior sales manager, senior local government officer)
  - Clerical (e.g. clerk, secretary)
  - Sales or services (e.g. commercial traveller, shop assistant, nursery nurse, care assistant, paramedic)
  - Foreman or supervisor of other workers (e.g. building site foreman, supervisor of cleaning workers)
  - Skilled manual work (e.g. plumber, electrician, fitter, train driver, cook, hairdresser)

- Semi-skilled or unskilled manual work (e.g. machine operator, assembler, postman, waitress, cleaner, labourer, driver, bar-worker, call centre worker)
- Other (please specify)
- Have never worked

Lifestyle and screening history

The following questions are about your lifestyle and your past screening decisions. Please answer as honestly as you can as this will help us with our analysis.

- **In general, would you say your health is**
  - Excellent
  - Very good
  - Good
  - Fair
  - Poor

- **What is your smoking status?**
  - Never smoked
  - Used to smoke
  - Smokes up to 20 per day
  - Smokes 20 or more per day

- **What is your height?**
  - ____ ft/m

- **What is your weight?**
  - ____ st/kg

- **Have you ever had cancer?**
  - Yes
  - No

- **Have your parents or any brothers or sisters ever had cancer?**
  - Yes
  - No
  - Don’t know/prefer not to answer

- **Have you been invited to take part in any of the following screening programmes?**
  (Please note that they are not offered to everyone). Information is in the format of (what it involves, who it is offered to)
  a. Abdominal aortic aneurysm screening (ultrasound, men aged over 65)
  b. Bowel cancer screening (poo sample or colonoscopy, men and women aged 60-74)
  c. Breast cancer screening (mammogram, women aged 50-70)
  d. Cervical cancer screening (smear test, women aged 25-64)
  - Yes
  - No
  - Not eligible

- **Which of the following screening programmes did you take up the invitation to take part in?**
  a. Abdominal aortic aneurysm screening (ultrasound, men aged over 65)
  b. Bowel cancer screening (poo sample or colonoscopy, men and women aged 60-74)
  c. Breast cancer screening (mammogram, women aged 50-70)
  d. Cervical cancer screening (smear test, women aged 25-64)
  - Yes
  - No
  - Not eligible

Thoughts and beliefs about cancer

The next section asks about your thoughts and beliefs about cancer. Please answer as honestly as you can as this will help us with our analysis. The questions are about YOUR opinion and so there is not a correct answer.

- **Below are some statements that are sometimes made about cancer. For each of these statements how much do you agree or disagree with them?**
  a. These days, many people with cancer can expect to continue with normal activities and responsibilities
  b. Most cancer treatment is worse than the cancer itself
  c. I would NOT want to know if I have cancer
  d. Cancer can often be cured
  e. Going to the doctor as quickly as possible after noticing a symptom of cancer could increase the chances of surviving
    - o Strongly agree
    - o Agree
    - o Neither disagree nor agree
    - o Disagree
    - o Strongly disagree

Developing targeted screening programmes by looking at risk factors for cancer

Because some types of cancers are rare, it would be very expensive to screen all adults in the UK and most of them would not benefit from the screening programme. One way of reducing the cost of screening and increasing the number of people who benefit is to only offer screening to people who we think are more likely to develop certain types of cancer.

A person's risk of developing certain types of cancer depends on many factors. These include age, sex, smoking, BMI/overweight and family history.

Please consider the following scenarios and answer the questions honestly. We would like to know YOUR opinion.

Scenario A
Imagine how you would feel in the following scenario…

Let's say you are feeling fine. You receive a letter inviting you to screening for a type of cancer as you have reached a certain age. The letter states that 'experts recommend that men get their first screening test at age 50 and women at age 60.' They then explain that 'the reason they recommend starting earlier in men is because this cancer is 2–3 times more common in men than women'.

- **How reasonable does it seem to you that experts recommend using age and sex to decide when to start screening?**
- **How reasonable does it seem to you that, in this scenario, men would be offered screening earlier than women?**
- **How comfortable are you with experts using age and sex to decide when you should start screening for this cancer?**
    - o Not at all reasonable (1)
    - o Not at all reasonable
    - o Not very reasonable
    - o Somewhat unreasonable
    - o Somewhat reasonable
    - o Very reasonable
    - o Extremely reasonable (6)

    - o Not at all comfortable – extremely comfortable (1-6)

Scenario B
Now imagine that you receive a different letter inviting you to screening. In that it describes how experts have developed a calculator to estimate how likely an individual is to develop a type of cancer, and to identify at what age you should first have screening for this cancer.

This calculator, which is based on scientific research studies, uses information available from routine GP records, such as your age, sex, BMI (body mass index, a measure that uses your weight and height to work out if your weight is healthy) and smoking status. You are also asked to fill out a questionnaire for the calculator,

which asks about your family history of cancer and your lifestyle, including how much physical activity you do and your diet.

Like the screening tests, it is not 100% reliable. Someone who is estimated to be at higher risk based on the calculator is not destined to get this cancer. A low risk estimate also does not mean someone is completely without risk of getting this cancer.

- **How reasonable does it seem to you that doctors would require you to fill out a questionnaire to predict your risk of this cancer?**
- **How comfortable would you be filling in the questionnaire and giving information about your family history and lifestyle?**
- **How reasonable does it seem to you that experts would use a calculator that includes these factors to predict each person's risk of this cancer and use that to decide what age to start screening for this cancer?**
- **How reasonable does it seem to you that people at higher risk based on this calculator would be invited to screening for this cancer at a younger age than people at lower risk?**
- **How comfortable are you with experts using a cancer risk calculator based on these factors to decide what age you should start screening for this cancer?**
- **How comfortable would you be about having to wait until you were older to start screening for this cancer if you were low risk based on this calculator?**
    - Not at all reasonable – extremely reasonable (1-6)

    - Not at all comfortable – extremely comfortable (1-6)

Scenario C
Having certain genes can also increase your risk of a type of cancer. Imagine that you are invited to be tested for these high-risk genes by providing a sample of blood or a cheek swab. This information would then be used to calculate how likely you are to develop this cancer and used to decide what age to start screening.

The relationship of these genes with the risk of this cancer is based on scientific research. Like the risk calculators above, the calculator based on genes is not 100% reliable. Someone who is estimated to be at higher risk based on their genes is not destined to get this cancer. A low risk estimate also does not mean someone is completely without risk of getting this cancer.

- **How reasonable does it seem to you that doctors would require you to provide a sample of blood or a cheek swab to predict your risk of this cancer?**
- **How comfortable would you be providing a sample of blood or a cheek swab to allow experts to test whether you have high-risk genes?**
- **How reasonable does it seem to you that experts would use a genetic test to predict each person's risk of this cancer and use that to decide what age to start screening for this cancer?**
- **How reasonable does it seem to you that people at higher risk based on their genes would be invited to screening for this cancer at a younger age than people at lower risk?**
- **How comfortable are you with doctors using a genetic risk calculator to decide what age you should start screening for this cancer?**
- **How comfortable would you be about having to wait until you were older to start screening if you were low risk based on a genetic risk calculator?**
    - Not at all reasonable – extremely reasonable (1-6)

    - Not at all comfortable – extremely comfortable (1-6)

Thank you for completing the survey. Your time and contribution are very much appreciated.

Supplementary File 2: Questionnaire 2 (completed at end of community jury)

Thoughts and beliefs about cancer

This section asks about your thoughts and beliefs about cancer. Please answer as honestly as you can as this will help us with our analysis. The questions are about YOUR opinion and so there is not a correct answer.

- **Below are some statements that are sometimes made about cancer. For each of these statements how much do you agree or disagree with them?**
  a. These days, many people with cancer can expect to continue with normal activities and responsibilities
  b. Most cancer treatment is worse than the cancer itself
  c. I would NOT want to know if I have cancer
  d. Cancer can often be cured
  e. Going to the doctor as quickly as possible after noticing a symptom of cancer could increase the chances of surviving
    - Strongly agree
    - Agree
    - Neither disagree nor agree
    - Disagree
    - Strongly disagree

Developing targeted screening programmes by looking at risk factors for cancer

Because some types of cancers are rare, it would be very expensive to screen all adults in the UK and most of them would not benefit from the screening programme. One way of reducing the cost of screening and increasing the number of people who benefit is to only offer screening to people who we think are more likely to develop certain types of cancer.

A person's risk of developing certain types of cancer depends on many factors. These include age, sex, smoking, BMI/overweight and family history.

Please consider the following scenarios and answer the questions honestly. We would like to know YOUR opinion.

Scenario A
Imagine how you would feel in the following scenario…

Let's say you are feeling fine. You receive a letter inviting you to screening for a type of cancer as you have reached a certain age. The letter states that 'experts recommend that men get their first screening test at age 50 and women at age 60.' They then explain that 'the reason they recommend starting earlier in men is because this cancer is 2–3 times more common in men than women'.

- **How reasonable does it seem to you that experts recommend using age and sex to decide when to start screening?**
- **How reasonable does it seem to you that, in this scenario, men would be offered screening earlier than women?**
- **How comfortable are you with experts using age and sex to decide when you should start screening for this cancer?**
    - Not at all reasonable (1)
    - Not at all reasonable
    - Not very reasonable
    - Somewhat unreasonable
    - Somewhat reasonable
    - Very reasonable
    - Extremely reasonable (6)

    - Not at all comfortable – extremely comfortable (1-6)

Scenario B
Now imagine that you receive a different letter inviting you to screening. In that it describes how experts have developed a calculator to estimate how likely an individual is to develop a type of cancer, and to identify at what age you should first have screening for this cancer.

This calculator, which is based on scientific research studies, uses information available from routine GP records, such as your age, sex, BMI (body mass index, a measure that uses your weight and height to work out if your weight is healthy) and smoking status. You are also asked to fill out a questionnaire for the calculator, which asks about your family history of cancer and your lifestyle, including how much physical activity you do and your diet.

Like the screening tests, it is not 100% reliable. Someone who is estimated to be at higher risk based on the calculator is not destined to get this cancer. A low risk estimate also does not mean someone is completely without risk of getting this cancer.

- **How reasonable does it seem to you that doctors would require you to fill out a questionnaire to predict your risk of this cancer?**
- **How comfortable would you be filling in the questionnaire and giving information about your family history and lifestyle?**
- **How reasonable does it seem to you that experts would use a calculator that includes these factors to predict each person's risk of this cancer and use that to decide what age to start screening for this cancer?**
- **How reasonable does it seem to you that people at higher risk based on this calculator would be invited to screening for this cancer at a younger age than people at lower risk?**
- **How comfortable are you with experts using a cancer risk calculator based on these factors to decide what age you should start screening for this cancer?**
- **How comfortable would you be about having to wait until you were older to start screening for this cancer if you were low risk based on this calculator?**
    - Not at all reasonable – extremely reasonable (1-6)

    - Not at all comfortable – extremely comfortable (1-6)

Scenario C
Having certain genes can also increase your risk of a type of cancer. Imagine that you are invited to be tested for these high-risk genes by providing a sample of blood or a cheek swab. This information would then be used to calculate how likely you are to develop this cancer and used to decide what age to start screening.

The relationship of these genes with the risk of this cancer is based on scientific research. Like the risk calculators above, the calculator based on genes is not 100% reliable. Someone who is estimated to be at higher risk based on their genes is not destined to get this cancer. A low risk estimate also does not mean someone is completely without risk of getting this cancer.

- **How reasonable does it seem to you that doctors would require you to provide a sample of blood or a cheek swab to predict your risk of this cancer?**
- **How comfortable would you be providing a sample of blood or a cheek swab to allow experts to test whether you have high-risk genes?**
- **How reasonable does it seem to you that experts would use a genetic test to predict each person's risk of this cancer and use that to decide what age to start screening for this cancer?**
- **How reasonable does it seem to you that people at higher risk based on their genes would be invited to screening for this cancer at a younger age than people at lower risk?**
- **How comfortable are you with doctors using a genetic risk calculator to decide what age you should start screening for this cancer?**
- **How comfortable would you be about having to wait until you were older to start screening if you were low risk based on a genetic risk calculator?**
    - Not at all reasonable – extremely reasonable (1-6)

    - Not at all comfortable – extremely comfortable (1-6)

<u>**Your experience of the community jury**</u>

- **Overall, the presentations by the experts…**
    a. Helped me understand the issues
    b. Were referred to during our deliberation
    c. Helped discussion between community jury members
    - Strongly disagree
    - Disagree
    - Undecided

- Agree
- Strongly agree

- *Additional comments*

- **Overall, the facilitator…**
  - **a. Helped to stimulate the group's reflections**
  - **b. Respected the opinions of participants**
    - Strongly disagree
    - Disagree
    - Undecided
    - Agree
    - Strongly agree

    - *Additional comments*

- **Overall, during the discussions between jurors…**
  - **a. I felt comfortable sharing my ideas**
  - **b. I could express disagreements**
  - **c. I remained engaged throughout the experience**
  - **d. Everyone was offered the opportunity to state their views**
  - **e. I considered there might be a difference between what I would want for myself, and what I would recommend for the community as a whole**
  - **f. I discussed my preferences and values in relation to the jury question**
  - **g. I considered the views of others during the deliberation**
    - Strongly disagree
    - Disagree
    - Undecided
    - Agree
    - Strongly agree

    - *Additional comments*

- **The outcomes of our jury…**
  - **a. Our community jury reached a clear recommendation/s**
  - **b. Our community jury's recommendation/s directly addressed the question we were given at the start**
  - **c. We discussed why we made our recommendations.**
  - **d. We made our recommendations for the community as a whole not just for ourselves.**
    - Strongly disagree
    - Disagree
    - Undecided
    - Agree
    - Strongly agree

    - *Additional comments*

- **Other comments (OPTIONAL): Do you have anything to add about your experience on this community jury?**
  - *Additional comments*

Thank you for completing the survey. Your time and contribution are very much appreciated.
